# Supplementary material for: The Puzzle of Metabolite Exchange and Identification of Putative Octotrico Peptide Repeat Expression Regulators in the Nascent Photosynthetic Organelles of Paulinella chromatophora
Source: Front Microbiol. 2020 Nov 27;11:607182. doi: 10.3389/fmicb.2020.607182 (PMC7729196; doi:10.3389/fmicb.2020.607182)
Supplement: Supplementary file 1 [file Data_Sheet_1.PDF]

Supplementary Figures

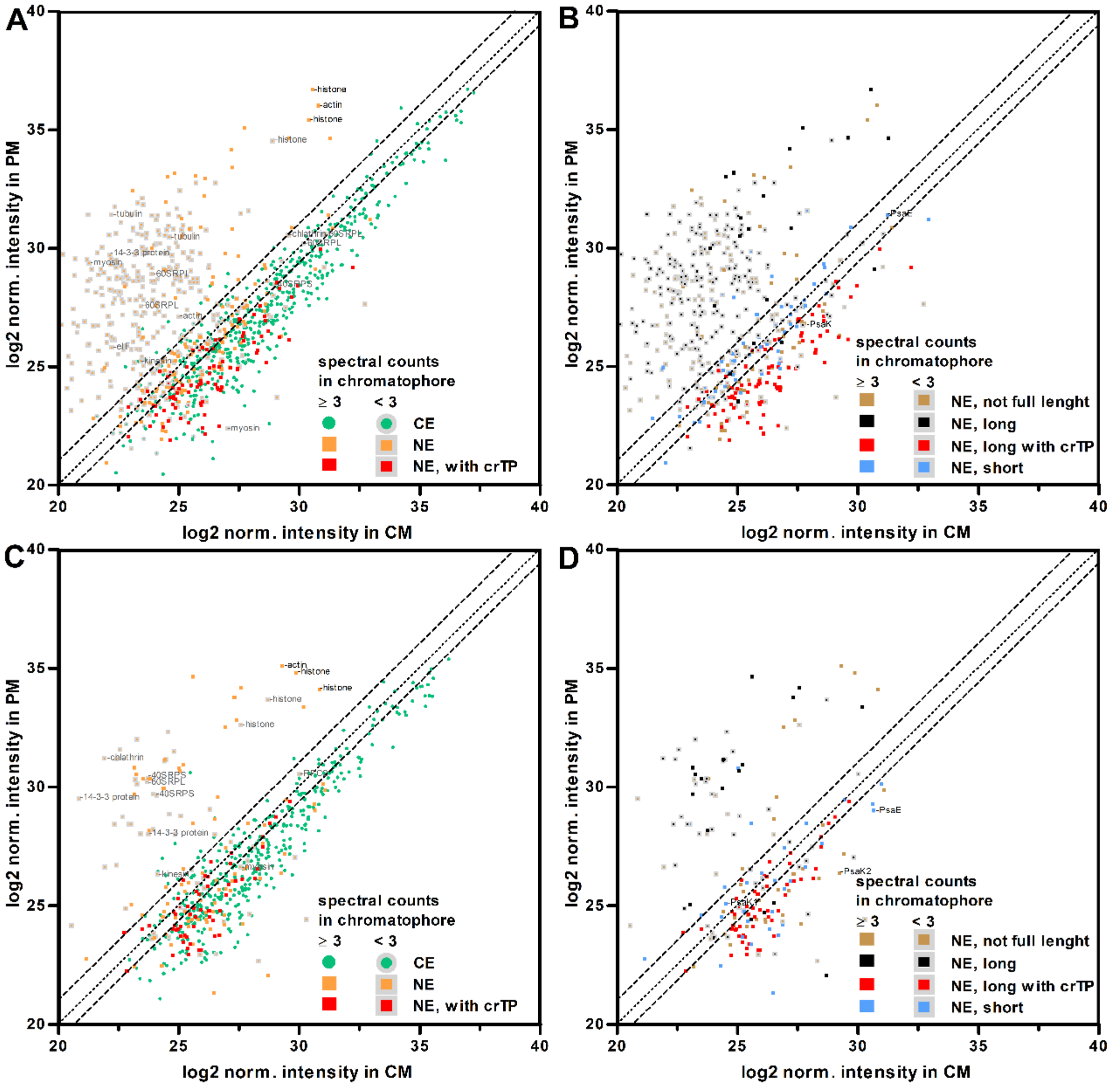

Supplementary Figure S1: Calibration of enrichment level thresholds for determination of import candidates. The $log2\ (\overline{normInt_{CM}})$ (see Methods, Protein enrichment analysis) was plotted against the $log2\ (\overline{normInt_{PM}})$ for proteins identified in CM and PM samples in MS experiment 1 **(A and B)** and MS experiment 2 **(C and D)**. CE, chromatophore-encoded; NE, nucleus-encoded; NE with crTP, nucleus-encoded proteins that contain a crTP; NE long, proteins longer than 125 aa; NE short, proteins shorter than 95 aa; NE not full-length, length and/or presence of crTP cannot be determined due to lacking sequence information (includes also proteins which lack an SL or an in-frame Stop-codon upstream to their putative translation start sites). Proteins identified with <3 SpC in the chromatophore (CM + CL samples) were not considered as import candidates. For the remaining proteins identified with ≥3 SpC in chromatophore samples, the enrichment factor in CM as compared to PM fraction $(\overline{normInt_{CM}}/\overline{normInt_{PM}})$ was calculated (see **Supplementary Tables S2** and **S3**). Proteins enriched in the chromatophore by a fold change of at least 1.5 in at least one out of two MS experiments are regarded "Enriched with high confidence"; proteins depleted in the chromatophore by a fold change <0.5 in at least one MS experiment are regarded "Depleted"; proteins with weak fold changes ≥0.5 but ≤1.5 (indicated by dashed lines) in both MS

experiments are regarded “Enriched with low confidence”. Proteins enriched in one experiment but depleted in the other are classified as “unclear”. Many of the proteins excluded from the analysis by using the ≥3 SpC-threshold represent highly expressed contaminating host-proteins like 40S/60S ribosomal proteins, histones, and cytoskeletal components (A and C) and lack a crTP (B and D). The “Enriched with low confidence” range includes several crTP-containing proteins and also chromatophore-encoded proteins (A and C) as well as short import candidates and small proteins involved in photosynthesis (B and D). The range was introduced to calibrate our experimental data based on our knowledge regarding the chromatophore localization of crTP-containing proteins, small proteins involved in photosynthesis, and chromatophore-encoded proteins.

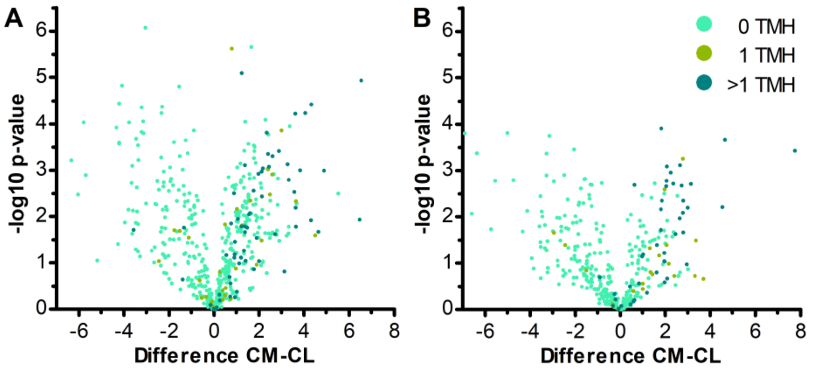

Supplementary Figure S2: Membrane proteins are enriched in chromatophore membrane fractions (CM) compared to chromatophore lysate (CL). Enrichment of chromatophore-encoded membrane proteins in **(A)** MS experiment 1 and **(B)** MS experiment 2. The number of predicted TMHs is indicated by a color code. The difference between the mean log2-transformed normalized intensities of individual proteins in CM and CL samples ($\overline{log2(normInt_{CM})} - \overline{log2(normInt_{CL})}$; Difference) is plotted against significance (-log10 p-values in Student´s t-test) for proteins identified in all three triplicates of either CM or CL or both. A positive difference value indicates protein enrichment in CM, negative values indicate depletion in CM compared to CL samples. Values for proteins detected only in one sample have been imputed and are only shown when their difference is significant.

61 nucleus-encoded group 1 proteins:

```
                            10        20        30        40        50        60        70        80        90       100
                    ....|....|....|....|....|....|....|....|....|....|....|....|....|....|....|....|....|....|....|....|..
scaf10537-m.74914   ----------------MVVGFLLPADANTN-VYVWGGAVAFISAIGLSAIV----LDKVINRTREGDIVEPTDPRYRAKSQFDRMNDNDPSTPF-------------
scaf11441-m.78969   ----------------MTLGDGGVCLVCNQ-NGIFVMGAVGAIATVVFKVG----TKLQQRRLIPLNPPTQDATTVDPLQPSVDSTSSVGLEEL-------------
scaf18064-m.104324  ----------------MGADKPDSFETFMN-GVGNVCLQFGAGLFLGMSWFINP-LRIWDSNRVLEASKGSKPSPLPSKGAGKVGEL--------------------
scaf49054-m.178992  ---------------------MGIDFHGPE-LISRTVAGAGCAYGMYWLYS----RKDQYTAEEQEFYSVSSFFKNKFKLPFSWPPWAGWFKGKDV-----------
scaf33572-m.147014  ------------------------MTAGQD-FVNLLFPTGKIAGVAAIGMITWF-MTKYSLKSANDPVPDQDDLYGGDNWWDHTSDNGQIGLWNQDK----------
scaf34704-m.149622  --------------------MVTLGDSEIH-LTVGQALAVVLLGAGAWVGAIVY-KTKAHPKKYTKKIPFTGEVDQYGNYKVDDPDSPPVAA---------------
scaf26107-m.128484  --------MGFDLSASGLIEEAKSILVGEH-GMYLLGGIIVFKVVKAGGIYMFA-RHRMKKRQEANDSVIVSDSVLPSEL---------------------------
scaf29584-m.137513  ----------------MVFAPADIPLWTIP-ISRTLQIVGLTTLMVGSGWIAGR-VQSYKAAEKHFREKEKSRAS--------------------------------
scaf11958-m.81185   --------------------------MGHD-PLGYAMLGFALTIFPLAFVA----SAKEYFYGSDTFKGNGTAHYRKDENRLEISPYEVTQDVEKLRAEREARGIFF
scaf12323-m.82733   ------------------MGAFEEWQEKLG-IPFHPAYLVLPAMLGVSLVAYLV-KTFREAIGVQDPYYQNECESNLWQPQTHPWDFSQPGTEPSI-----------
scaf14182-m.90232   -----------MVADLLGLAQSTSDVMKSN-SAVLWFSGALGVVGIAGFTWAFM-RPNDGSASFPEGVPGPGGFGTAQHKPPPE-----------------------
scaf14591-m.91815   ----------------MLFGPGGGPGGPPE-IDNITAGGLIVIMFASTAGLWIT-AQQALTQVDPSVIDPFNYFT--------------------------------
scaf16155-m.97626   ---------------------------MDT-LQYVTVGSSLVMGAGVLLWT----KNFYSHYIEHGGTVSTIRKEMEKEMEEAEASSHRGNSL--------------
scaf16238-m.97925   ----------------MAVDPELPEINIGV-TILDAVIVANISFLLMYGSLSML-GRAARKILKTGKIETHLDLNTGEIIKTFTRNIKPTTITAPAT----------
scaf16791-m.99896   ---------------------------MED-VNYLAVAEMFVGMTMVHISYAMI-RNQSKYADTEETKDLPWDSEGKSPYPKDSTVLW-------------------
scaf17508-m.102386  --------------------------MSLE-VPPLALASFGITAAGIGMVLYYV-WQDRQLLAEDAVDVEDKTKAGQSKLPGA------------------------
scaf17524-m.102436  --------------------------MISE-QTSTIILLGLFGMGLAAIPL----YQRRADREAKEFKENMERAQLAKLDKKTKKELNL------------------
scaf17649-m.102892  ------------------MTTIIEAHPEFR-TAVVGVSVALSAGLSSLYAYAVY-KWIEEGFKKKDGVNKDTL----------------------------------
scaf18129-m.104557  -----------MVWHVGHPVPELTPHELFK-QAAMIGVFALPGLFYAQE------QRTERRKLFRDKDPKARQGRASPKDISDFENLF-------------------
scaf19030-m.107575  -----------MMLGPNGDPPQPLSHRIEQ-VAALVVTAGLTIVSYWLFFS----WAQGRGDIPAQQPLPPQVAELHKSRPR-------------------------
scaf19852-m.110196  ------------------------MDVDFG-AIFYFLTFGGLVGGAFFMMNQGV-GASKDMMNMDRRSQQRKGLKPEVGGTFDAEFERQRLEKAFNDS---------
scaf20187-m.111259  ---------------------MLLYPAADP-VMILCTSALVIGVAAGVAVG----VDKHFNKMPKSDSLEAELSREMKKRNLQRI----------------------
scaf20970-m.113655  ----------------MVSDNPFINYITSP-QFWLVNMGYIALYLGFRGLI----EDNVDDRNRRGGTGHSLGPDGPVEG---------------------------
scaf21270-m.114594  ------------------MIFDENGENKYG-LAGTLILGFVAVSVAGIVVPMIS-NRIGPNAQHYTTEDGGRVTVNDTGSFMDTARSAPQL----------------
scaf21554-m.115432  ----------------MVLDVPADTYTFAG-PFTYLAWDVILTGGLAGTIYTLY-NMSKPVNERISAGPPNQPTVSQGGIFKGNVGAGSEYL---------------
scaf21859-m.116371  ---------------------------MTD-YTGMLVAMGGLTALALGGTVFML-RGSDEGIDARRARMNRPPLPKNAPGAPANEE---------------------
scaf22570-m.118457  ------------------------MVPIDG-LTAQLMVGGGLGVVGFGVIYFMA-GAGMNQDDDLDLFTGGETPEQVEAKPFVKKEGKKKKKGG-------------
scaf22658-m.118727  ----------------MVNFPSAMYDVQNM-GMMTIALSLATGVGVGAWVL----KQASTPYEEPEHEDFLDSIL--------------------------------
scaf24345-m.123581  ---------------MGLLTSEVEVVDQYA-WWKTAAAVLLLILWAGMAGKAIG-EALLDDAGKQDSLEGNAAPPTSSNTK--------------------------
scaf25032-m.125510  ---------------------MEIDLLKTG-LTVLEVSSVIGAVVFGVAYSYGT-SKFQESQFNARKAREAIDYLTTKLPTV-------------------------
scaf26247-m.128866  ----------MVLDQYINAGHAMTQDDIIR-YTGYLAAAGGVLTIINTIYSF---KTSDNSQAGMGDVKDFMFKEGRDPKPPARK----------------------
scaf26682-m.130062  -----MVQIVLDEAEKRATVSFGGQCRDFT-VPAIVAGGAAFIGVLFFPRLTSI-LALLGMSGKCVPTSNGTEAPLTIKLVGFDDLLGSPTQR--------------
scaf28437-m.134580  ----------MLAEVMVVSDMSSNIDRVRA-MSSLGWQASIVFAAVSTAGVVYM-NKDNDNFFAKQLFDNIKKYWGEK-----------------------------
scaf28627-m.135056  --------------------------MVET-SLNIALPMIGVISGVFGLIMVAG-SRGGGGGGGGDSYSSYVPPVPPPTARGGYTWNK-------------------
scaf29101-m.136255  ----------MVLDQYINAGHAMTQDDIIR-YTGYLAAAGGVLTIINTIYSF---KTSDNSQAGMGDVKDFMFKEGRDPKPPARK----------------------
scaf29281-m.136705  ------------------MYEAVDTPLVSP-GFMAALITYTVPLVAIGAGVWKF-REGQQMVARIEARLEREAQKKDLKQPP-------------------------
scaf30821-m.140533  ----------------MAKSNQNPELGEGP-SIFVMLALIQGGLTAFCGGCAFL-YCTIRLALENAGQVHDITEDEDSPASMQKRYGAVPAPIM-------------
scaf31918-m.143184  ---------------------MTAGVSEPV-FWPIALAMAGGIAAFVSWST----TDQYVEGRDRANKKPTFVNENEKYYDR-------------------------
scaf33120-m.145970  ---------------------MNELGSDDY-VFWSPVPVIIASGAIIFAMLVTS-PFRIRAPPPREENPIPEPGEPSID----------------------------
scaf33246-m.146268  ------------MLEVCDAWGTVCVSLGTT-YIPIGLPSIIGAGVCTLPAIFAF-QQLFKTQRERDEAVSKLEELERRTGPKT------------------------
scaf34225-m.148553  ---------------------MGGNIFTDF-LFGAACGVGGLLTVWSMGILGPG-MRGAHTEKLHTSEGKYSDVPYDPGAEFVKRFRRDDLGQWEEPT---------
scaf35692-m.151791  -------------------MTAQLSNQAGD-YTTPILFVSIYFGSMLYWAW----KLNDKFRRRTELPMRKKLQESDDPFRRVYDDF--------------------
scaf38478-m.157842  ----------------MVDIFGLDSLTPTQ-AKQFGGFLVGLAGLYIVLFTGRA-VRELNNDRLKISEVTRDSLTRPQDR---------------------------
scaf38561-m.158043  ----------------MLLAEEAIHNPIPL-VQSLMALNVVGLGSLVVAVGWTV-YRVRQDNLGEDSGPTAPGPVVKSRPRKTDFREL-------------------
scaf41653-m.164469  ---------------------MIDLHTPSA-MLFETNAAFLSVLGLYLV------KRNPGAASMKEEIRKRREAEEKK-----------------------------
scaf44176-m.169563  ------------------MIFDENGENKYG-LAGTLILGFVAVSVAGIVVPMIS-NRIGPNAQHYTTEDGGRVTVNDTGSFMDTARSAPQL----------------
scaf47746-m.176463  ----------------MGMDFFVSEGVVQW-PHVIGAAVIGASSIGLPALVLWV-ETPRKELGEKRRNYGFIFKN--------------------------------
scaf50116-m.181040  ---------MALEEEWEIGAPSSRPSMWAR-ALALVILLGTVVPSIVLPIQMAI-DDHKYHKFVKEHPELAKQIDERLAKAQAQTQ---------------------
scaf51627-m.184009  ---------------------MDFGDHHDG-VAYIFTTGLFAAFFINIR------KQIQFARKKENLLKRSRRRFWQDGGTTDAFFDDQPQAGSS------------
scaf52394-m.185473  ----------MFEREDEELEKLTDTQYSIV-AALTGVAGIATGLMLKTCFTAPL-KVLLGSPSLPSLLQQAQANGVFPS----------------------------
scaf52582-m.185802  ---------------------MLESLPKND-MIMLGLAIAASYGFYAYG------RNMSARNAALQSDDEDGLSWQRKEPSNPQTPQKPRTTGRGGF----------
scaf12228-m.82351   --------------------------MANA-ALFALGSGLLALGMFTRFHVVVV-DRDEDIQEIRSKIREDGFDVDKFIKDREKKL---------------------
scaf16759-m.99777   ---------------------MPSQTLLSN-RWAAAAAATTLALGALLWVGLG--RRKRLRSLAAWYLPASLPHPPDHPNQSHPHRPVNNRELATLGIEYST-----
scaf19203-m.108122  ---------------------MAIDQAIFK-TITWRLIAIGISGTASFAITGNY-KASLGIIGSQTIAGSVIYYGHEKAWEKIKDQMDIHEAASQSSSSSV------
scaf26143-m.128583  -----MKNFGRVGGRRFPASTGGDPEPSDD-FPAFLTGVACTLATGAIVAACML-PPRREAYTPEPVVYEYSEIITIYDY---------------------------
scaf27338-m.131785  ---------------------------MKD-FYTIPVAIGVVSAFALGGIPLLS-MMLSSRSEEKSNAARAAAAAAGEEPGKVVGGMFPPQDSADWAKQFRKK----
scaf27898-m.133262  ---------------------MVHHVYWVS-NIYQGFMIATVGTFGTLGLILYN-EMGPRIGYPKDEPRRRKEDYPNEPPPPNRFGTSGRNRGR-------------
scaf2825-m.30676    ---------------------------MNG-LLAWWGQLLALLLGPRSFLV----SRSKPAGQASYRLAEGAEWRSTFWTACES-----------------------
scaf32507-m.144601  MHEFQTDAWRFIQQVADPQIQLPATCGSRK-VPLWLASAAFILGVIIVPRWTAI-VATGSSIKCPIQGLVPSEPKPYYHARNWQ-----------------------
scaf35590-m.151586  ----------------MMGTVPWHETQLFK-GFVLGIGISLGTALWRGIGF----VMTPASVLLREKIDCATEKVRAHLGTPHIQRKAPQYR---------------
scaf37177-m.154986  [illegible]
```

204 nucleus-encoded group 2 proteins:

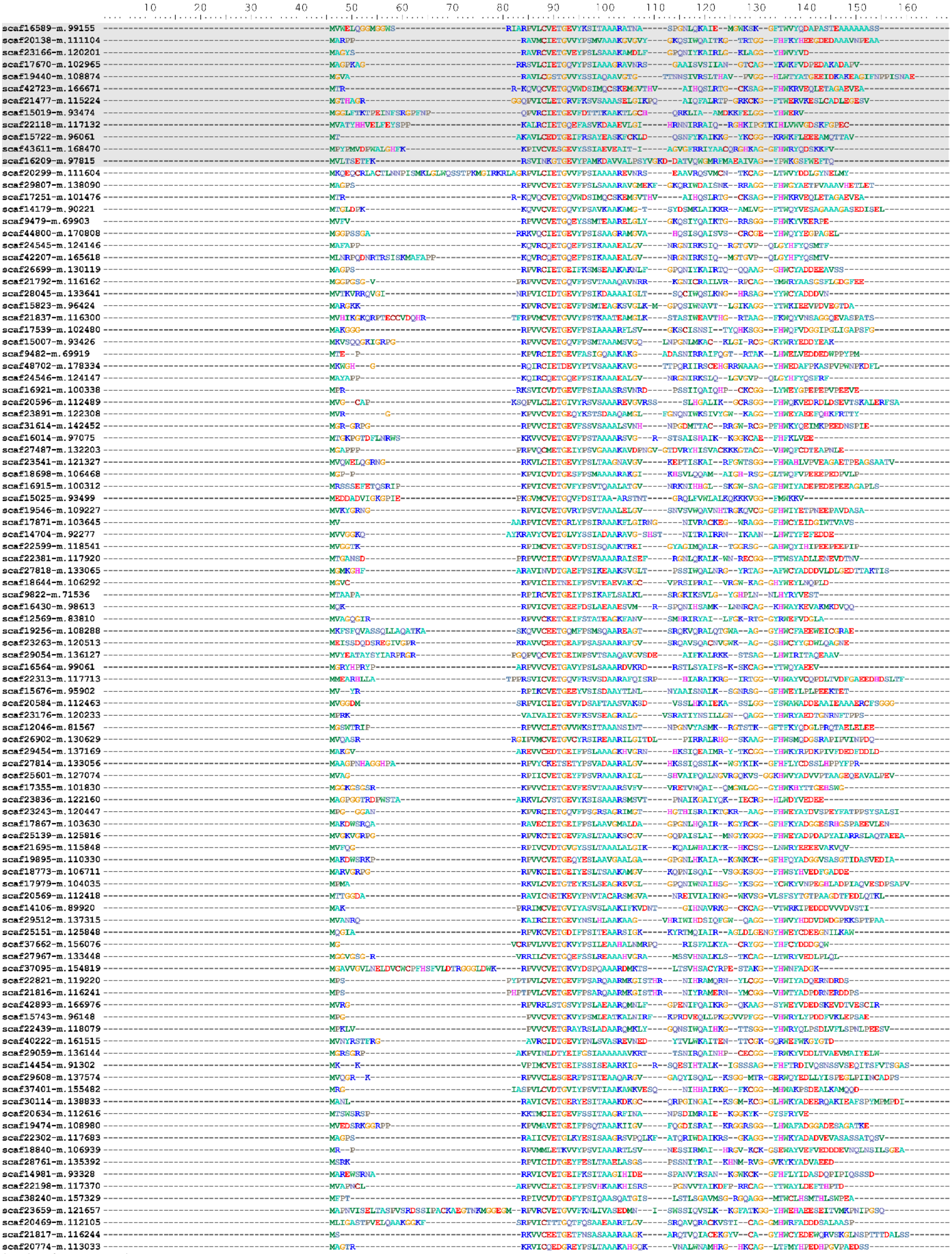

(continued on next page)

204 nucleus-encoded group 2 proteins continued:

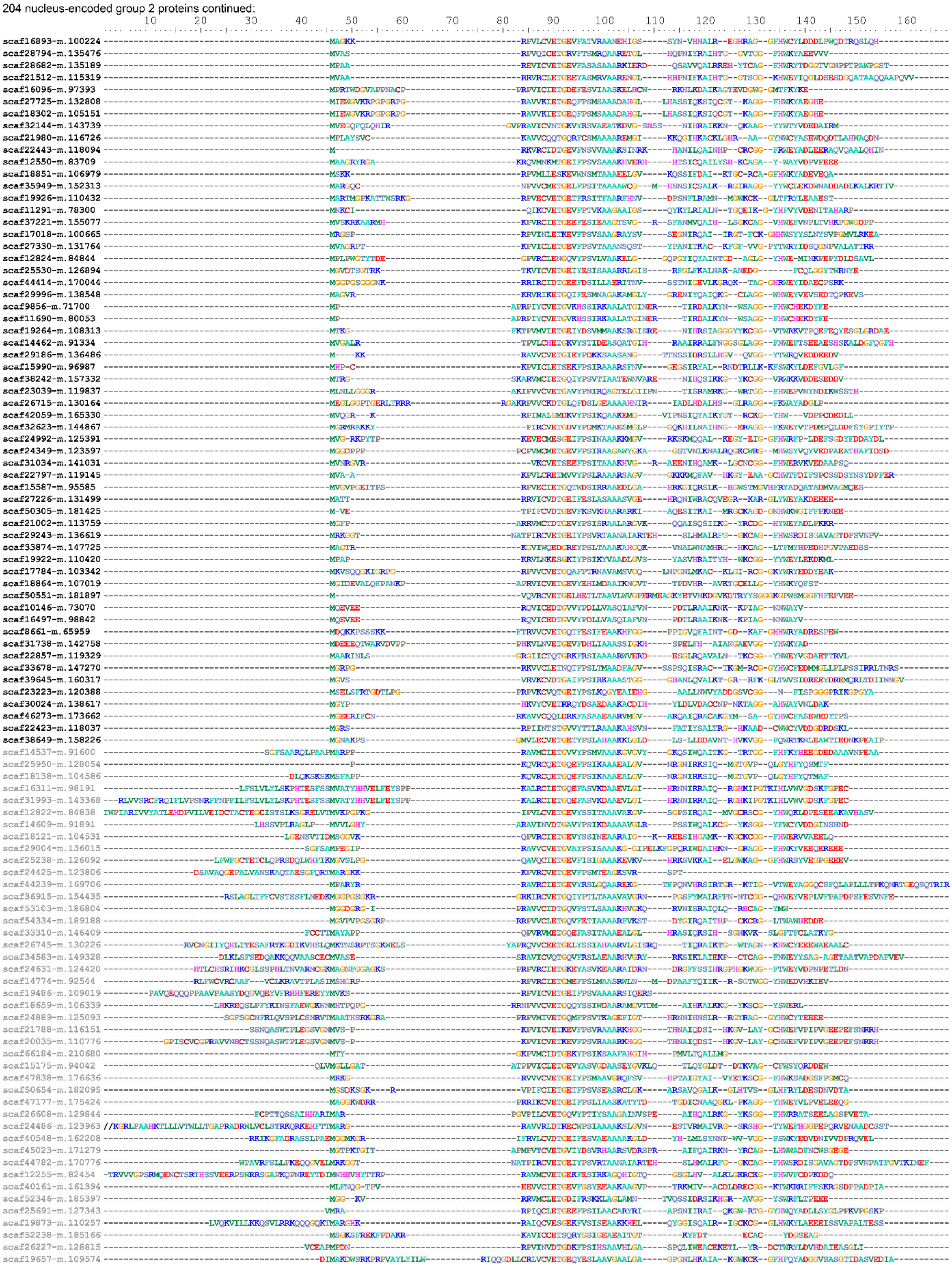

38 nucleus-encoded group 3 proteins:

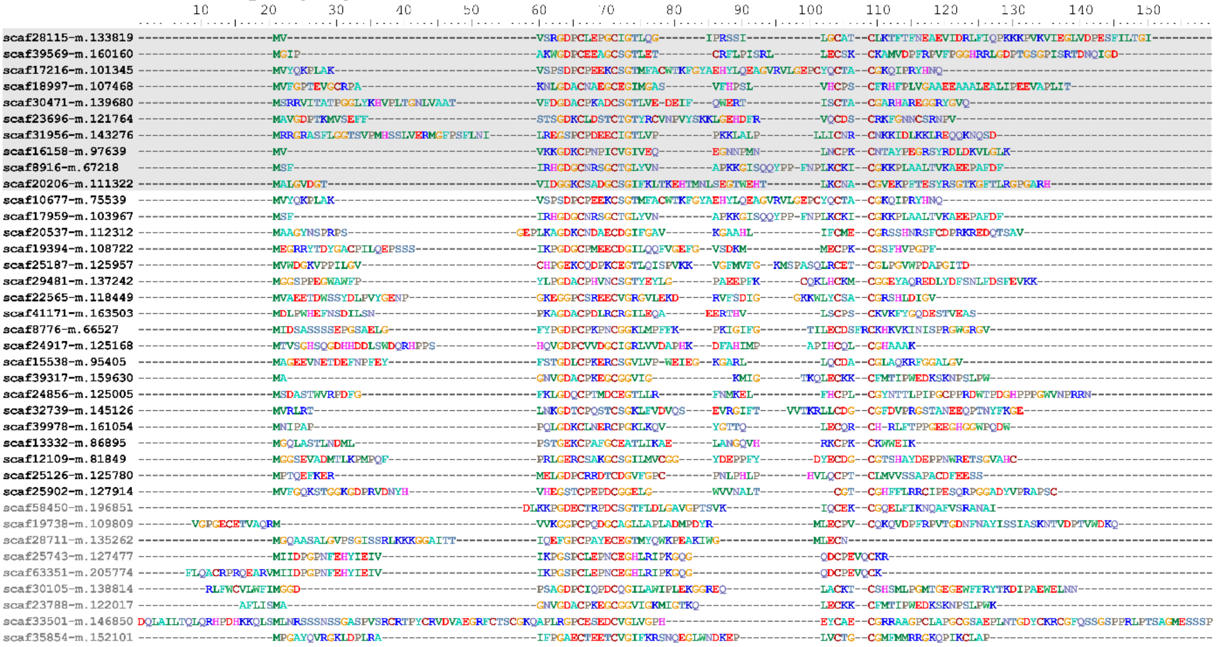

Supplementary Figure S3: Alignments of potential further group 1 to 3 short import candidates identified in the *P. chromatophora* transcriptome. Translated transcripts representing short proteins with a predicted TMH in the N-terminal 2/3 of the sequence that contains >20% small aa (Gly, Ala, Ser) and feature an N-terminus with a net charge ≤0 were regarded as putative further group 1 proteins. Translated transcripts that show significant similarity (FIMO, $p<e^{-08}$ or $p<2e^{-07}$) to the conserved motifs in MS-identified group 2 or group 3 proteins (highlighted in grey in **Fig. 4A**) were regarded as putative group 2 and 3 proteins, respectively. 161 and 29 of the proteins are short (<90 aa), respectively. For the remaining proteins either the start methionine could not be identified unequivocally (as a spliced leader or an in frame stop codon upstream of the start ATG are missing in the corresponding transcript) and/or a stop codon is missing at the C-terminus (grey sequence titles). Thus, their length cannot be determined. Importantly, none of the identified proteins belonging to either group is unequivocally long (>250 aa).

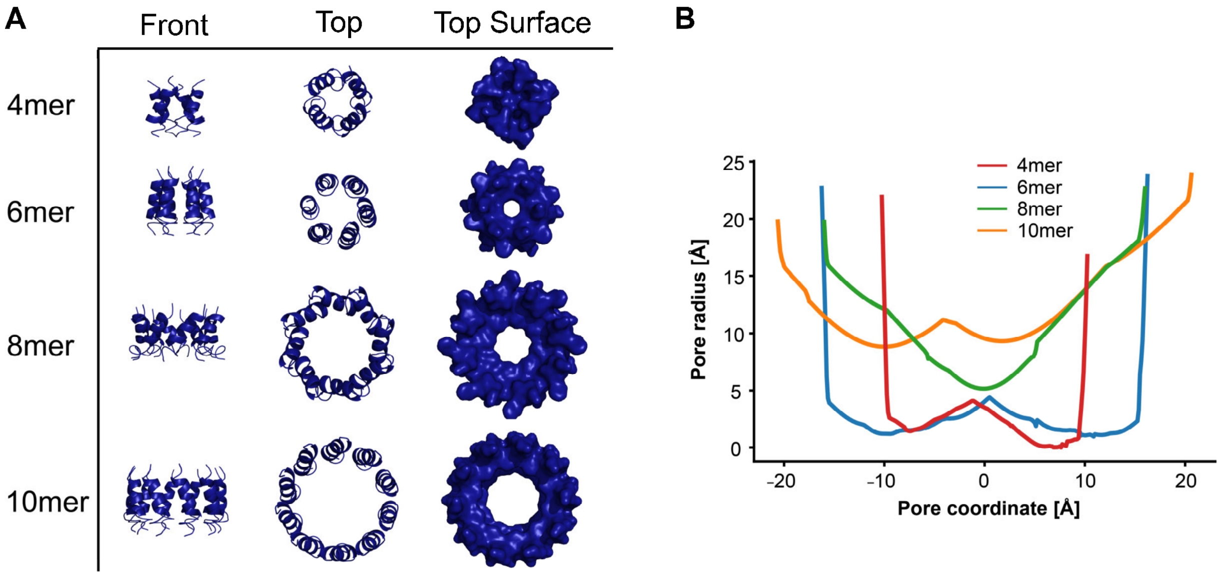

Supplementary Figure S4: Hypothetical *n*-mer pore-like assemblies for scaffold29584-m.137513. (A) The predicted TMH of caffold29584-m.137513 was extracted from a homology model made with TopModel (Mulnaes et al. 2020), primarily based on the cryo-EM structure of the bovine respirasome (PDB ID 5LUF). The structure was arranged in 4, 6, 8, and 10mer assemblies with CHDOCK (Yan and Huang 2019); the best scoring assembly for each case is shown from the front and top; for the latter, also the molecular surface is shown. According to pore geometry, structures with six or more helices [i.e. of higher order than the predicted stoichiometries (**Table S4**)] might allow permeation of metabolites through the pore, as suggested from previous studies (Grice et al. 1997). **(B)** Radius along the pore of structures shown in panel (A) as calculated with HOLE (Smart et al. 1996). For reference, the bottleneck radius of common metabolite transporters such as multidrug and Omp channels is close to the radius of a water molecule (1.4 Å) (Khalid et al. 2007, Khalid and Sansom 2006, Song et al. 2014, Vaccaro et al. 2008). An increasing pore size begs the question of how such a channel could selectively transport metabolites, while maintaining the electrochemical gradient of the chromatophore. VDAC, in the OM of mitochondria, remains partially selective despite having a pore of 13 Å radius due to a stochastic gating mechanism (Berezhkovskii and Bezrukov 2018) which might be considered as a model for metabolite transport across the chromatophore IM.

Supplementary References

Berezhkovskii, A.M., and Bezrukov, S.M. (2018). Stochastic gating as a novel mechanism for channel selectivity. Biophys J. 114, 1026-1029.

Grice, A.L., Kerr, I.D., and Sansom, M.S.P. (1997). Ion channels formed by HIV-1 Vpu: A modelling and simulation study. FEBS Lett. 405, 299-304.

Khalid, S., Holyoake, J., and Sansom, M.S.P. 2007. Molecular Dynamics Studies of Membrane Proteins: Outer Membrane Proteins and Transporters. In: Biophysical Analysis of Membrane Proteins.

Khalid, S., and Sansom, M.S.P. (2006). Molecular dynamics simulations of a bacterial autotransporter: NalP from *Neisseria meningitidis*. Mol Membr Biol. 23, 499-508.

Mulnaes, D., Porta, N., Clemens, R., Apanasenko, I., Reiners, J., Gremer, L., Neudecker, P., Smits, S.H.J., and Gohlke, H. (2020). TopModel: Template-based protein structure prediction at low sequence identity using top-down consensus and deep neural networks. J Chem Theory Computation. 16, 1953-1967.

Smart, O.S., Neduvelil, J.G., Wang, X., Wallace, B.A., and Sansom, M.S.P. (1996). HOLE: A program for the analysis of the pore dimensions of ion channel structural models. J Mol Graphics. 14, 354-360.

Song, J.N., Ji, C.G., and Zhang, J.Z.H. (2014). Insights on Na^+ binding and conformational dynamics in multidrug and toxic compound extrusion transporter NorM. Proteins: Struct Funct Bioinf. 82, 240-249.

Vaccaro, L., Scott, K.A., and Sansom, M.S.P. (2008). Gating at both ends and breathing in the middle: conformational dynamics of TolC. Biophys J. 95, 5681-5691.

Yan, Y., and Huang, S.-Y. (2019). CHDOCK: a hierarchical docking approach for modeling Cn symmetric homo-oligomeric complexes. Biophys Rep. 5, 65-72.
